# Working-Class Members of Hospital Committees

**Published:** 1900-03-24

**Authors:** 


### WORKING-CLASS MEMBERS OF HOSPITAL COMMITTEES.

A good deal has lately been said at Cardiff, in the newspapers and otherwise, about the representation of the working men subscribers on the Infirmary Board. It is a subject which is of interest in many towns besides Cardiff, and it is one which may easily be manipulated to the detriment of the charities concerned unless it is dealt with on fair give and take lines.

The difficulty arises from the fact that, sugar it over as we like, there is a strong difference of interests between the two classes of subscribers—those on the one hand who give as a charity to others, and have no prospect of ever getting any personal return for their money, except, of course, the pleasure of helping to maintain a useful institution, and the gratification of being able by the distribution of tickets to assist their poorer neighbours, and those, on the other hand, who give with the definite intention of providing a refuge for men of their own class, and even for themselves if they should happen to be ill. Look at it as we like, and admire the working men as much as we will for their liberality and their readiness to help each other in their difficulties, it is impossible to blink the fact that their charity is a sort of casting bread upon the waters.

It is this trace of the co-operative principle which places "shop collections" on a totally different plane from ordinary guinea subscriptions, and is the reason and excuse for the special arrangements made in so many hospitals for the representation of the working-men subscribers on the boards of management. These arrangements, necessary as they are, can easily be so represented as to bear the aspect of a grievance. For example, it is not uncommon to find a rule to the effect that the workmen in any shop, or the members of any trade association subscribing to the funds of the hospital, may nominate as a governor one of their number for every, say, £10 contributed during the preceding year, and that these governors may appoint from among themselves a certain number to represent them on the committee of management. This is a fair and simple arrangement, which offers very marked advantages in certain respects to the working class subscribers. Nevertheless the class distinction drawn by it is apt to be resented, and the fact that while a rich man's guinea makes him a governor, it takes nearly ten guineas to make a governor out of a working men's representative, is capable of being put in such a way as to rankle in the bosom of the independent Briton and to create a sense of injustice in his mind. Of course, any such feeling is founded upon a misconception. So far is it from being true that the "shop collections" are unfairly dealt with, the truth lies rather in the opposite direction. Indeed, we should be inclined to say that there is far more danger of hospitals being captured by the organised trade subscribers and used for class purposes than there is of these trade subscribers finding any difficulty in pressing home their views as to hospital management. In some places this is a very real danger.

We do not know whether the question of the power of a hospital to refuse subscriptions has ever been seriously raised. In any case we can hardly conceive of such a right being put in force for any length of time, and there is but little doubt that by a little finesse, although acting strictly within the rules, it would be possible for the working-men subscribers to gain a large, if not a predominating, degree of control over the management of many hospitals. If, instead of sending in the whole of their 20 or 30 guineas as the subscription of such and such a shop, or such and such a trade, they were, after making their collections, to hand a guinea to Smith, a guinea to Jones, and a guinea to Robinson, and so on as far as the guineas went round, telling each of these persons to become a guinea subscriber, Smith, Jones, and Robinson, and all the rest of them would become governors, and each of them would be as good in voting power as my lord the president. Moreover, accustomed as shop leaders always are to act in concert and in accordance with strict trade discipline, they would vote as one man, and could obtain a far greater amount of power than would be represented by the mere amount of their subscriptions. This is by no means an imaginary risk. In the management of a hospital it does not generally matter very much whether the leading influence is church or chapel, radical or conservative, town or county. There are plenty of good men on every side and in every party who will administer such a charity ably and impartially. But it does seriously matter that the leading influence should be exercised by governors who are "representatives" and feel bound to act in the interests of those by whom they are returned and by virtue of whose subscriptions they hold their seats. It is by no means difficult to imagine circumstances in which, without any intentional dishonesty—in fact, in the honest endeavour to carry out the mandate of those by whom they had been put in power—such governors and such members of committees might be led to divert the hospital from its proper object by giving preference to those by whom, as the saying would be, "the charity was supported." If working-class "representative" governors were to become a numerous and powerful body, it would be difficult to prevent "charity" being ousted by "co-operation," and one can hardly doubt that, under such circumstances, the unorganised poor and the unrepresented sick would go to the wall.

